# Magnitude and predictors of unfavorable management outcome in surgically treated patients with intestinal obstruction in Ethiopia: a systematic review and meta-analysis

**DOI:** 10.1186/s12893-023-02017-3

**Published:** 2023-05-16

**Authors:** Fentahun Adane, Megbar Dessalegn

**Affiliations:** 1 Department of Biomedical Sciences, School of Medicine, Debre Markos University, Debre Markos, Ethiopia; 2 Department of Surgery, School of Medicine, Debre Markos University, Debre Markos, Ethiopia

**Keywords:** Intestinal obstruction, Magnitude, Management, Poor outcome, Predictors, System Review, Meta-analysis, And Ethiopia

## Abstract

**Background:**

Unless an emergency surgical intervention is conducted, intestinal obstruction may result in high morbidity and mortality. In Ethiopia, the magnitude and predictors of unfavorable management outcomes in surgically treated patients with intestinal obstruction are highly variable and inconsistent. The aim of this study was; therefore, to estimate the overall prevalence of unfavorable management outcome and its predictors among surgically treated patients with intestinal obstruction in Ethiopia.

**Method:**

We searched articles from databases from June 1, 2022, to August 30, 2022. Cochrane Q test statistics and I^2^ tests were applied. We used a random-effect meta-analysis model to overcome the impact of heterogeneity among the included studies. In addition, the association between risk factors and unfavorable management outcome in surgically treated patients with intestinal obstruction was investigated.

**Results:**

This study included a total of twelve articles. The pooled prevalence of unfavorable management outcome in surgically treated patients with intestinal obstruction was 20.22% (95% CI: 17.48–22.96). According to a sub-group analysis by region, Tigray region had the highest prevalence of poor management outcome, which was 25.78% (95% CI: 15.69–35.87). Surgical site infection was the most commonly reported symptom of poor management outcome (8.63%; 95% CI: 5.62, 11.64). The length of postoperative hospital stays (95% CI: 3.02, 29.08), duration of illness (95% CI: 2.44, 6.12), presence of comorbidity (95% CI: 2.38, 10.11), dehydration (95% CI: 2.07, 17.40), and type of intraoperative procedure (95% CI: 2.12, 6.97) were all significantly associated with unfavorable management outcome of intestinal obstruction among surgically treated patients in Ethiopia.

**Conclusion:**

According to this study, the magnitude of unfavorable management outcome was high among surgically treated patients in Ethiopia. Unfavorable management outcome was significantly associated with the length of postoperative hospital stays, duration of illness, comorbidity, dehydration, and type of intraoperative procedure. Medical, surgical and public health measures are pivotal to reduce unfavorable management outcome in surgically treated intestinal obstruction patients in Ethiopia.

**Supplementary Information:**

The online version contains supplementary material available at 10.1186/s12893-023-02017-3.

## Background

Intestinal obstruction (IO) is the term used to describe a complete or partial obstruction to the passage of intestinal contents. It is a potentially dangerous surgical emergency because there is a high rate of morbidity and mortality [1]. It is a typical surgical emergency requiring prompt diagnosis in addition to immediate, sensible, and efficient care [2]. It is a significant contributor to deaths, financial costs, and admissions to emergency surgical units in hospitals all over the world [3, 4].

The prevalence of IO is known to be high in India, Iran, Afghanistan, and a few African countries, including Ethiopia. It has been the leading cause of the acute abdominal disorders in Africa [5–8]. Various studies indicate that IO accounts roughly for 49–60% of all cases of surgically treated acute abdominal disorder in Ethiopia [9–11].

Intestinal obstruction (IO) is classified as small bowel obstruction (SBO) or large bowel obstruction (LBO) based on its anatomical location [12]; it can also be mechanical or functional based on the underlying pathophysiology of obstruction [13]. SBO caused by adhesions, strangulated hernia, malignancy, and volvulus has all been implicated in the etiology of IO [14]. The causes of IO vary according to population and location. Hernia and volvulus are the most common causes of IO in the developing world, whereas adhesions are the most common in the developed world. However, these established patterns are changing in Africa [15–18].

Although management of intestinal obstruction has improved as a result of the development of more sophisticated diagnostic tests and imaging techniques, the condition remains a major public health concern, particularly in developing countries [9, 19, 20] where Ethiopia is not an exception. Regardless of its underlying causes, a surgery for IO sometimes lead to a variety of postoperative complications. It is a difficult problem determined by numerous patient-related and clinical-related factors resulting in complications such as surgical site infection, wound dehiscence, leakage, pneumonia, and sepsis. Many of these unfavorable management outcomes could be avoided if the factors associated with the surgical treatment outcome of intestinal obstruction are predetermined and all necessary precautions are taken before and after the procedure [21, 22]. The outcome of disease management may be a good indicator of how well a country’s surgical services are performing. Several factors contribute to IO patients’ poor outcomes. Poor health-seeking behavior, ignorance, poverty, and poor clinical judgment are some of these risk factors [17, 23].

Additionally, the factors that influence unfavorable treatment outcome in surgically treated patients with intestinal obstruction in Ethiopia as well as the postoperative complications differ from district to district. Although a few studies have been reported, their results are inconsistent requiring synthesis of the available data. The purpose of this study was to determine the overall magnitude of unfavorable treatment outcome and associated risk factors in surgically treated patients with intestinal obstruction in Ethiopia which could provide a glimpse in to the understanding of the associated epidemiological and clinical data important for policy makers.

## Methods

### Protocol registration

The protocol for this study can be found at (https://www.crd.york.ac.uk/prospero/# my Prospero) with an identification number CRD42022358662.

### Search strategy

The search strategy attempted to find both published and unpublished studies. Electronic databases, conference proceedings, websites, dissertations, and direct contact with the authors were used to gather information. A preliminary original search of PubMed, Science Direct, Google scholars, MEDLINE (Ovid) and CINAHL (EBSCO) was conducted on June 1, 2022, and was updated on August 30, 2022. The last search was carried out on August 30, 2022. The text words in the titles and abstracts of relevant papers, as well as the index keywords used to characterize the articles, were analyzed and used to build a thorough search strategy in partnership with a faculty librarian. The databases searched include MEDLINE (Ovid), PsycINFO (EBSCOhost), EMBASE (Ovid), CINAHL (EBSCOhost), Web of Science (Direct access), Scopus (Direct access), JBI EBP database (Ovid) and African Journals Online (AJOL). The search strategy’s index phrases (topic headings) and keywords were customized to each database. To locate further studies, the reference lists of all identified relevant studies and systematic reviews were searched. Google scholar, Mednar, ProQuest, and dissertation databases were also used to look for unpublished studies. To obtain the most recent estimate, articles published in English from January 2015 to August 2022 were considered. The search words were specified for a comprehensive search that included all fields in records, as well as Medical Subject Headings (MeSH terms) to broaden the scope of the search in a PubMed advanced search. We combined keywords with the “OR” operator in the Boolean operator within each axis and then linked the search techniques of the two axes to the “AND” operator. The search terms were “magnitude” OR “epidemiology” AND “favorable treatment outcome” OR “unfavorable” AND “Ethiopia”. The definite searching detail in PubMed with MeSH terms was Magnitude[All Fields] AND predictors[All Fields] AND unfavorable[All Fields] AND (“treatment outcome“[MeSH Terms] OR (“treatment“[All Fields] AND “outcome“[All Fields]) OR “treatment outcome“[All Fields]) AND (“surgical procedures, operative“[MeSH Terms] OR (“surgical“[All Fields] AND “procedures“[All Fields] AND “operative“[All Fields]) OR “operative surgical procedures“[All Fields] OR “surgically“[All Fields]) AND treated[All Fields] AND (“patients“[MeSH Terms] OR “patients“[All Fields]) AND (“intestinal obstruction“[MeSH Terms] OR (“intestinal“[All Fields] AND “obstruction“[All Fields]) OR “intestinal obstruction“[All Fields]) AND (“Ethiopia“[MeSH Terms] OR “Ethiopia“[All Fields] OR Ethiopia* [All Fields]) were used (Table 1).

#### Study selection and outcome

Following the search, all citations found were compiled and imported into EndNote V20 (Clarivate Analytics, PA, USA). After deleting duplicates, two researchers (FA and MD) assessed all of the original search titles and abstracts against the predefined inclusion criteria. The two reviewers (FA and MD) separately reviewed the entire text of chosen citations against the inclusion criteria. The reasons for rejecting articles were documented and reported. Disagreements among the reviewers were settled through discussion. The study inclusion process and search results were reported in compliance with the Preferred Reporting Items for Systematic Reviews and Meta-analyses (PRISMA) guidelines [24–26].

**Table 1:** Example of MEDLINE/PubMed and Google Scholar database searches to determine the pooled magnitude and predictors of unfavorable treatment outcome in surgically treated patients with intestinal obstruction in Ethiopia, 2022

Sources	Search Engine	Number of studies
PubMed	MeSH terms was Magnitude[All Fields] AND predictors[All Fields] AND unfavorable[All Fields] AND (“treatment outcome“[MeSH Terms] OR (“treatment“[All Fields] AND “outcome“[All Fields]) OR “treatment outcome“[All Fields]) AND (“surgical procedures, operative“[MeSH Terms] OR (“surgical“[All Fields] AND “procedures“[All Fields] AND “operative“[All Fields]) OR “operative surgical procedures“[All Fields] OR “surgically“[All Fields]) AND treated[All Fields] AND (“patients“[MeSH Terms] OR “patients“[All Fields]) AND (“intestinal obstruction“[MeSH Terms] OR (“intestinal“[All Fields] AND “obstruction“[All Fields]) OR “intestinal obstruction“[All Fields]) AND (“Ethiopia“[MeSH Terms] OR “Ethiopia“[All Fields], OR Ethiopia* [All Fields]).	44
Science Direct	((unfavorable management outcome OR favorable management outcome and predictors OR Associated factors) AND Ethiopia AND (incidence OR prevalence OR magnitude))	36
Google scholar	A combination of the above key terms (favorable management outcome, unfavorable management outcome, intestinal obstruction, operative surgical procedures, prevalence, magnitude, predictors, associated factor, Ethiopia)	52
Medline		8
Embase		6
JBI database		6
AJOL		2
Manual search		4
Research repositories		2
Total retrieved articles		160
Finally full articles relevant to our review		12

NB. African Journals Online (AJOL)

### Eligibility criteria

#### Inclusion criteria

Articles on the magnitude and predictors of unfavorable management outcome in surgically treated patients with intestinal obstruction in Ethiopia were considered.

##### Study area

The articles which were conducted in Ethiopia were considered.

##### Study design

In Ethiopia, all observational studies (cross-sectional, case-control, and cohort) with original data on the magnitude, and predictors of unfavorable management outcome in surgically treated patients with intestinal obstruction were examined.

##### Language

Literature that was written in the English language.

##### Population

Studies that have been considered among surgically treated patients with intestinal obstruction in Ethiopia.

##### Publication condition

Both published articles and unpublished studies were considered.

##### Exclusion criteria

Unpublished and internet-inaccessible studies were excluded. We also excluded studies whose corresponding authors did not respond to our email inquiry for missing important data. Furthermore, after two reviewers (FA and MD) read the entire article, study that did not produce the desired result was omitted.

## Data extraction

All necessary data were extracted in Microsoft Excel TM using a checklist data extraction format created by two authors (FA, and MD). Using the checklist, the two authors extracted data from each of the original articles independently. The data extraction format for the magnitude of unfavorable management outcome was developed based on the first author, the location of the study, the publication year, the sample size, and the magnitude of unfavorable management outcome specified for the target population.

The data extraction format for predictors was modified for each predictor (duration of illness, length of hospital stays after surgery, comorbidity, dehydration, and intraoperative procedure). These variables were chosen by the authors because they appeared most frequently as associated factors in the studies that were included in this analysis. In this systematic review and meta-analysis, additional variables were considered as risk factors if they were examined as risk factors in two or more studies. To compute the odds ratio, two researchers (FA and MD) gathered data from the primary studies in the form of two-by-two tables for each identified risk factor.

### Outcome measurements

This systematic review and meta-analysis yielded two major findings. The primary outcome was the magnitude of unfavorable management outcome in surgically treated patients in Ethiopia with intestinal obstruction. The secondary outcome of the study was the predictors of unfavorable management outcome in surgically treated patients with intestinal obstruction in Ethiopia. The magnitude was determined by dividing the number of participants with unfavorable management outcome by the total number of surgically treated patients with intestinal obstruction in the study (sample size) and multiplying the result by 100.

### Quality assessment

The researchers (FA & MD) evaluated the quality of the articles included in this study using the Newcastle-Ottawa Scale adapted for cross-sectional study quality rating [27]. The tool is divided into three sections, the first of which, with five stars, assesses each study’s methodological excellence. The tool’s second section evaluates study comparability and assigns two points. The final section, which can be rated out of three stars, evaluates the original articles’ statistical analysis uniformity. The tool was used as a checklist to evaluate the overall quality of the primary articles. Using the tool as a checklist, the two authors independently assessed the quality of each of the original articles. Any disagreements among the authors about the quality evaluation results were resolved through discussion. The articles in this study range in quality from medium to high (7 out of 10 stars).

### Statistical analysis

The necessary data were extracted from Microsoft Excel TM and analyzed in STATA Version 15.0. The original studies were displayed as forest plots and tables. The authors calculated the standard error magnitude for each original article using the binomial distribution method. The use of test heterogeneity x^2^, I^2^, and p-values revealed heterogeneity in the prevalence of studies that were recorded [28]. According to the statistical analysis mentioned above, there were significant differences between the studies (I^2^ = 70.2%, p-value < 0.001). To estimate the combined effect of Der Simonian and Laird, a random effect meta-analysis method was used. Additionally, a univariate meta-regression model using the sample size and year of publication was used to determine the most likely source of heterogeneity, but none of the outcomes were statistically significant. At a 5% significant level, Begg’s regression intercept and Egger’s correlation tests were used to objectively inspect for potential publication bias [27, 29]. Additionally, publication bias was evaluated using Egger’s weighted regression and Begg’s rank correlation test methods (P > 0.05), which showed that it was statistically insignificant. Furthermore, subgroup analysis based on the regions in which the studies were conducted was carried out to reduce the random discrepancies between the point estimates from the primary study.

## Results

### Search results

**Fig. 1:**
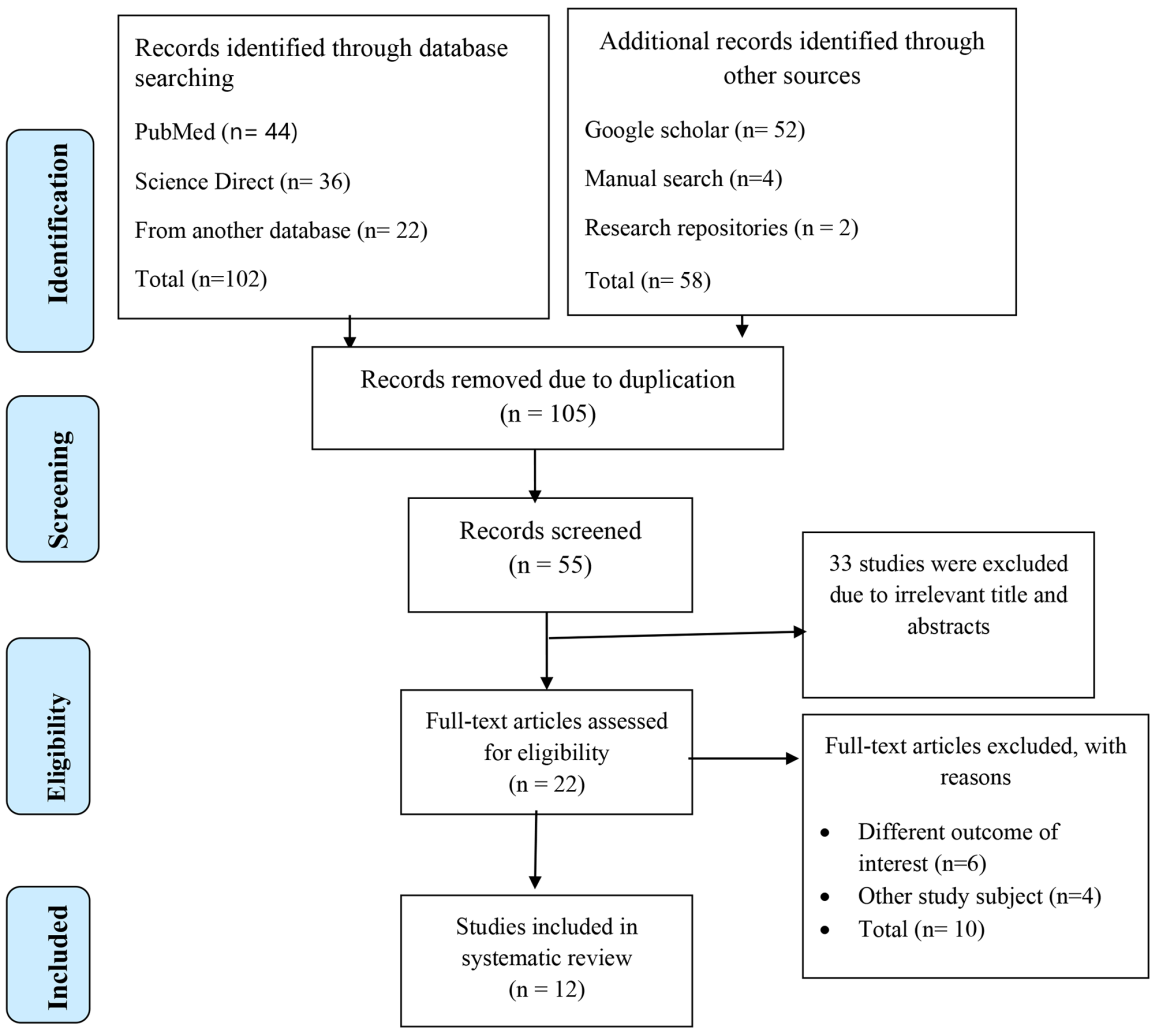
Flow diagram describing the selection of studies for the systematic review and meta-analysis of prevalence and predictors of poor management outcomes in surgically treated patients in Ethiopia (showing how articles were identified, screened, and included in the studies), 2022

The databases Medline (Pub Med), EMBASE, Science Direct, HINARI, Cochrane Library, Google Scholar, and other sources produced 160 publications on the magnitude and predictors of poor management outcome in surgically treated patients in Ethiopia. One hundred five articles were removed from the preliminary records because they were redundant. The remaining 55 articles’ titles and abstracts were scrutinized, and 33 were deemed irrelevant and removed. Following that, the remaining 22 full-text papers were obtained and evaluated for eligibility according to the predetermined criteria, leading to the exclusion of 10 articles, mostly as a result of the research population [30–33] and outcome of interest [34–39] being ineligible. Each study review’s quality score ranged from 7 to 9 out of a possible 10 points; thus, no studies were excluded based on this criterion. Finally, the final meta-analysis included twelve studies (Fig. 1).

### Characteristics of original articles

Twelve eligible original studies were finally included in this study. The studies were conducted between 2015 and 2022. The cross-sectional designs were used in all of the included studies. The pooled prevalence of magnitude and predictors of poor management outcome in surgically treated patients in Ethiopia were assessed in this study, which included 2,748 study participants. The studies were conducted in the Amhara [12, 40, 41], Oromia [23, 42, 43], Sothern nation nationalities and Peoples of Ethiopia (SNNP) [44–47], and Tigray [10, 48] regions. The sample sizes ranged from 135 in the study done at Amhara region [41] to 309 in another study conducted in the SNNP [44] (Table 2).

**Table 2:** Descriptive summary of twelve studies reporting the magnitude and predictors of unfavorable management outcome (cases) in surgically treated patients in Ethiopia included in the systematic review and meta-analysis, 2022

Author	Publication Year	Region	Sample Size	Case	Quality score (10 pts)	Prevalence with 95%
Ademe et al.,[40]	2021	Amhara	216	39	8	18.10 (12.97, 23.23)
Atalay et al.,[44]	2021	SNNP	309	69	9	22.30 (17.66, 26.94)
Batebo et al.,[45]	2022	SNNP	230	53	9	23.00 (17.56, 28.44)
Derseh et al.,[42]	2021	Oromia	254	54	9	21.30 (16.26, 26.34)
Fentahun et al.,[41]	2021	Amhara	135	29	8	21.50 (14.57, 28.43)
Gebre.,[10]	2016	Tigray	166	34	8	20.50 (14.36, 26.64)
Gebrie et al.,[46]	2019	SNNP	171	29	8	17.00 (11.37, 22.63)
Girma et al.,[47]	2021	SNNP	258	35	9	13.60 (9.42, 17.78)
Hagos.,[48]	2015	Tigray	299	92	7	30.8 (25.57, 36.03)
Jemere et al.,[23]	2017	Oromia	221	34	9	15.40 (10.64, 20.16)
Mariam et al.,[12]	2018	Amhara	227	38	9	16.70 (11.85, 21.55)
Soressa et al.,[43]	2016	Oromia	262	62	9	23.70 (18.55, 28.85)

### Meta-analysis

#### The magnitude and predictors of poor management outcome of intestinal obstruction

**Fig. 2:**
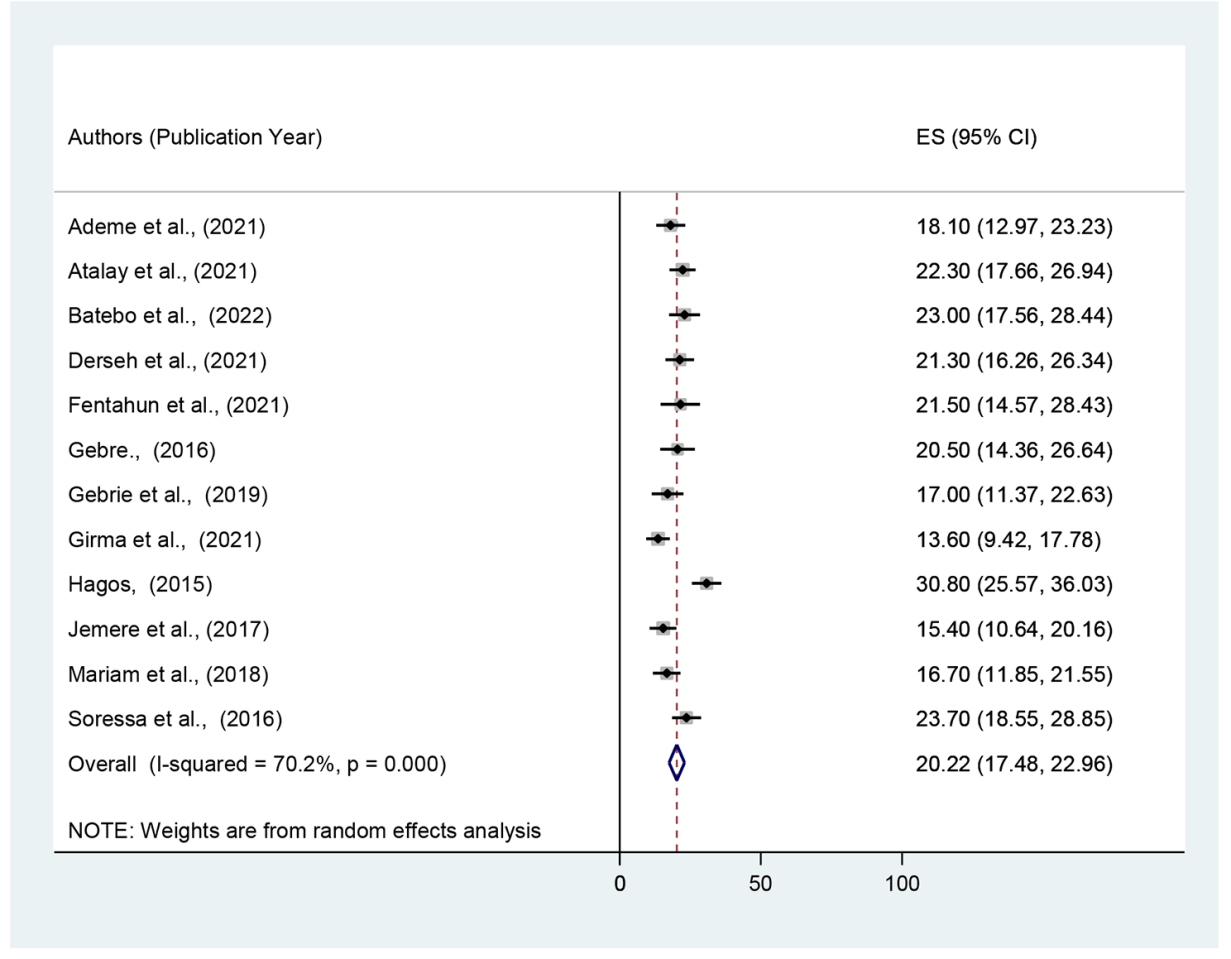
Forest plot of the pooled magnitude of unfavorable management outcome of intestinal obstruction in in Ethiopia, 2022

The pooled magnitude of unfavorable management outcome among surgically treated patients was 20.22% (95% CI: 17.48–22.96) (Fig. 2). However, significant heterogeneity was found across the studies, as disclosed by I^2^ statistic (I^2^ = 70.2%, p-value *<* 0.001). A random effect model was used to assess the pooled prevalence of unfavorable management outcome in surgically treated patients in Ethiopia. A univariate meta-regression model was also used to identify potential sources of heterogeneity by taking publication year and sample size into account. However, none of these variables were found to be statistically significant. There was no statistically significant publication bias, according to Beggs and Eggers’ tests (P value > 0.05).

#### Sub-group analysis

**Fig. 3:**
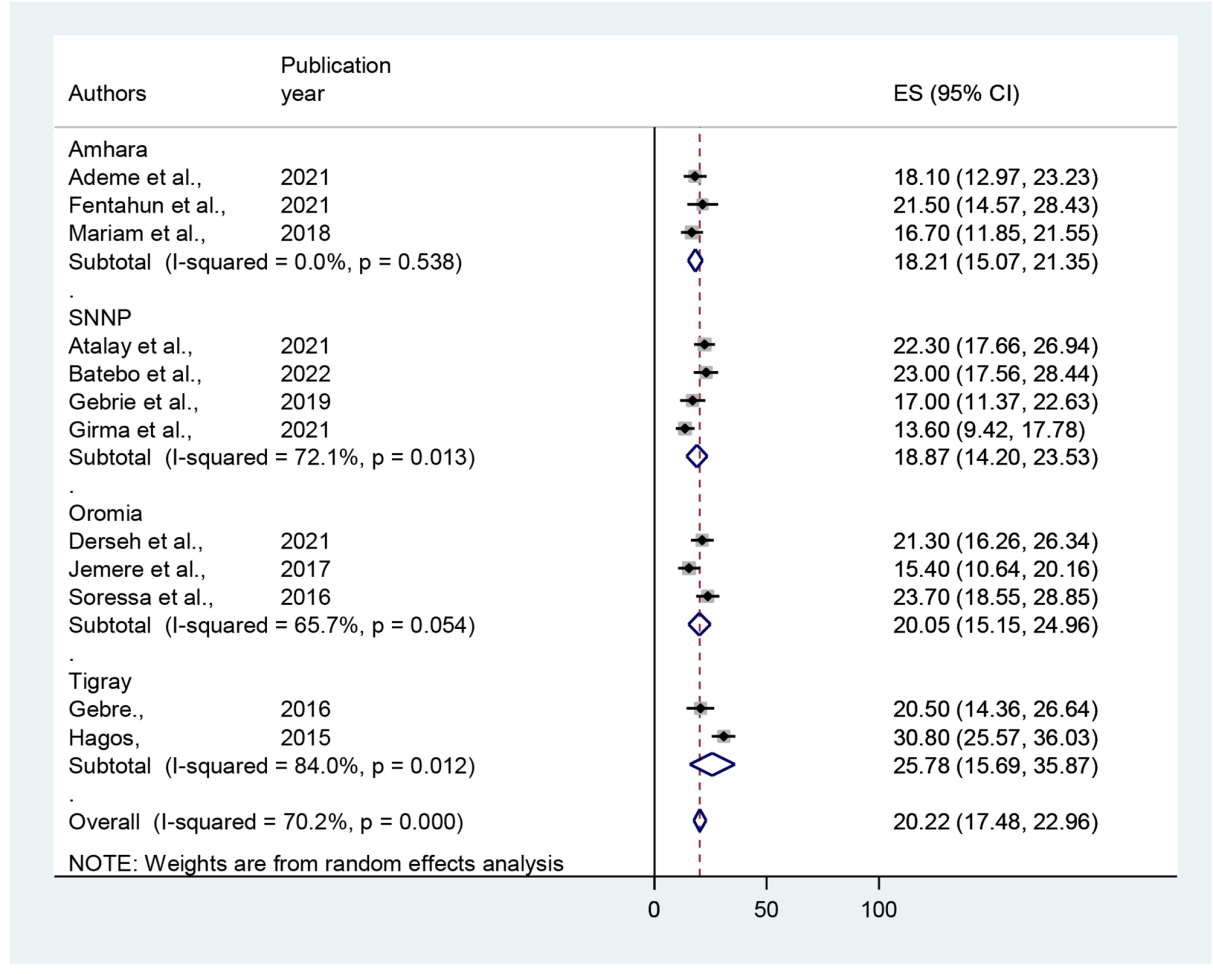
Forest plot of the sub - group analysis of prevalence of unfavorable management outcome of intestinal obstruction in different regions in Ethiopia, 2022

Due to significant heterogeneity among the publications included in this study, a region-based sub-group analysis was performed to investigate the likely cause of heterogeneity among the studies. The sub-group analysis shows the highest prevalence was observed in the Tigray region with a prevalence of 25.78% (95% CI: 15.69–35.87) followed by the Oromia region 20.05% (95% CI: 15.05–24.96) and SNNP region 18.87% (95% CI: 14.20-23.53) while the lowest prevalence was observed in Amhara region 18.21% (95% CI: 15.07–21.35) (Fig. 3).

#### Symptoms of unfavorable management outcome of intestinal obstruction

**Fig. 4:**
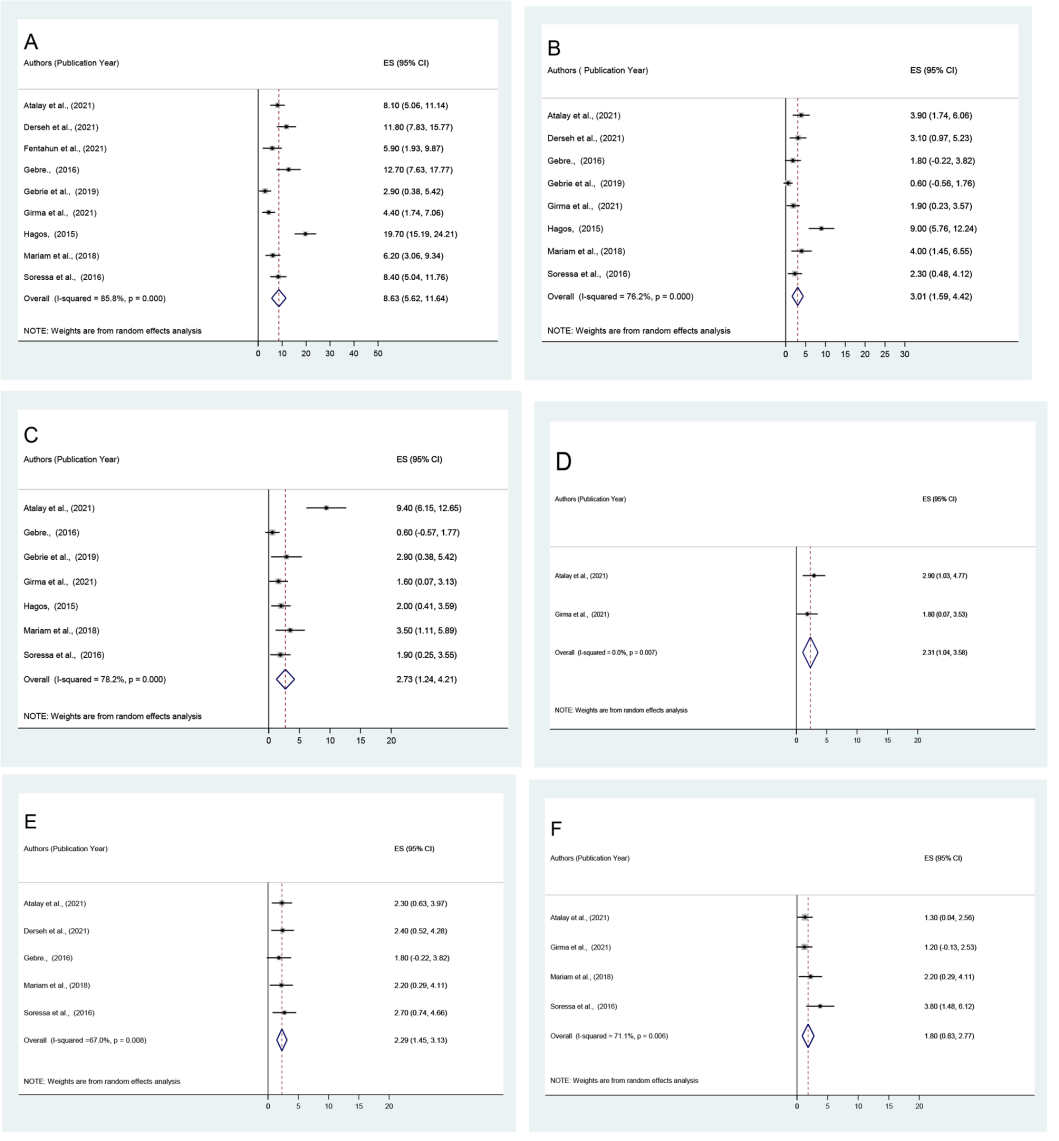
Forest plot depicting the pooled prevalence of symptoms of unfavorable management outcome of intestinal obstruction among surgically treated patients;( **A**: surgical site infection, **B**: pneumonia, **C**: septic shock, **D**: intraabdominal collection, **E**: anastomotic leak, and **F**: facial dehiscence) in Ethiopia, 2022

The most commonly reported types of unfavorable management outcome of intestinal obstruction were surgical site infection (8.63%; 95% CI: 5.62, 11.64), followed by pneumonia (3.01%; 95% CI: 1.59, 4.42), septic shock (2.73%; 95% CI: 1.24, 4.21), intraabdominal collection (2.31%; 95% CI: 1.04, 3.58), anastomotic leak (2.29%; 95% CI: 1.45, 3.13) and fascial dehiscence (1.80%; 95% CI: 0.83, 2.77) (Fig. 4: **A-F and** Table 3).

**Table 3:** The prevalence of types of unfavorable management outcome of intestinal obstruction among surgically treated patients in Ethiopia, 2022

No	Type of unfavorable management outcome of IO	Prevalence (95% CI)
1	Surgical site infection	8.63% (5.62, 11.64)
2	Pneumonia	3.01% (1.59, 4.42)
3	Septic shock	2.73% (1.24, 4.21)
4	Intraabdominal collection	2.31% (1.04, 3.58)
5	Anastomotic leak	2.29% (1.45, 3.13)
6	Facial dehiscence	1.80% (0.83, 2.77)

#### Predictors of unfavorable management outcome of intestinal obstruction

There was a significant association between the length of postoperative hospital stays (95% CI: 3.02, 29.08), duration of the illness (95% CI: 2.44, 6.12), presence of comorbidity (95% CI: 2.38, 10.11), dehydration (95% CI: 2.07, 17.40), and intraoperative procedure of resection and anastomosis (95% CI: 2.12, 6.97) and the unfavorable management outcome of intestinal obstruction among surgically treated patients in Ethiopia.

**Fig. 5:**
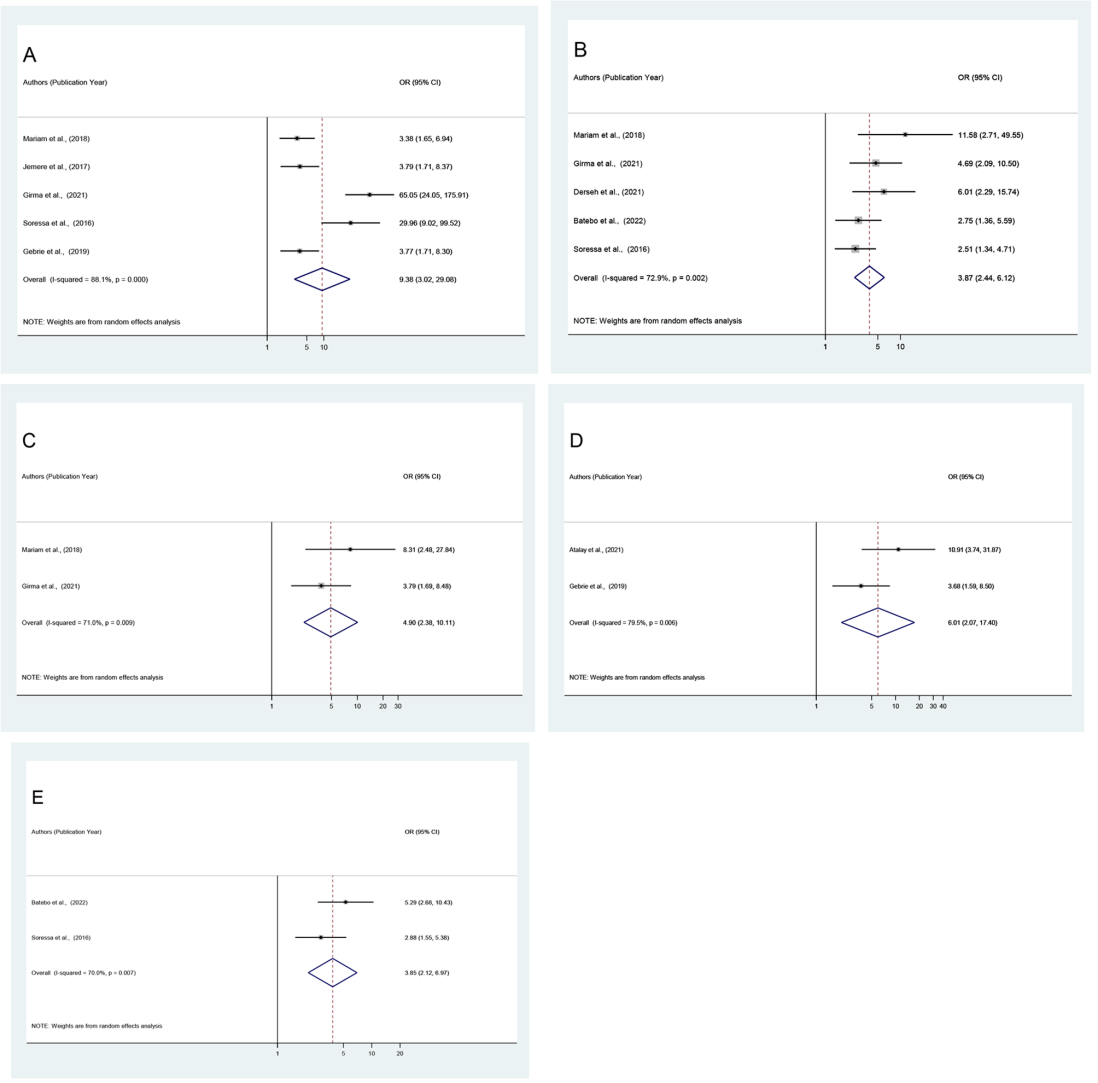
Forest plot depicting pooled odds ratio (log scale) of the associations between prevalence of poor management outcome and its predictors (**A**: length of hospital stays after surgery, **B**: Duration of illness, **C**: Comorbidity, **D**: Dehydration, **E**: Intraoperative procedure) in Ethiopia, 2022

In this study, surgically treated patients who stayed in the hospital for more than 8 days after surgery were 9.38 times more likely to experience an unfavorable management outcome than their counterparts (OR = 9.38 [95% CI: 3.02, 29.08]). Similarly, patients who arrived at the facility more than 24 h were approximately 3.87 times more likely to develop unfavorable management outcome (OR = 3.87, [95% CI: 2.44, 6.12]) and patients who presented with comorbidity were 4.90 times in odds of developing unfavorable management outcome (OR = 4.90, [95% CI: 2.38, 10.11]). Similarly, patients who had dehydration were 6.01 times more likely to have a poor management outcome than those who did not have dehydration (OR = 6.01, [95% CI: 2.07, 17.40]). Finally, in terms of the type of intraoperative procedure, patients who had resection and anastomosis had a 3.85 times greater risk of developing an unfavorable surgical management outcome (OR = 3.85, [95% CI: 2.12, 6.97]) (Fig. 5: A-E).

## Discussion

Surgical management of intestinal obstruction may have unpredicted pleasant or bad outcomes. More importantly, poor surgical care can cause significant harm to the patient [42]. This study aimed to determine the pooled magnitude of unfavorable management outcomes among surgically treated patients in Ethiopia and their associated risk factors.

In this study, the overall magnitude of unfavorable management outcome among surgically treated patients in Ethiopia was 20.22% comparable to studies conducted in Nigeria [49] and India [50] which found that poor management outcome had magnitudes of 20.77% and 25.89%, respectively. However, it is lower than the prevalence reported in studies from Nigeria (65.5%) [49] and Canada (64%) [51]. Though the observed prevalence of poor management outcome in this study was greater than the research findings in Nigeria (10%) [49] and Kenya (13.6%) [52]. The above discrepancies may be explained by differences in sociocultural, economic, and lifestyle patterns between nations, or by variations in statistical parameters such as sample size, overall study area infrastructures, internal hospital setups, and the knowledge and expertise of the medical staff regarding the diagnosis and treatment of intestinal obstruction.

 The present study sub-group analysis result revealed that the pooled magnitude of unfavorable management outcome among surgically treated patients in Ethiopia varies across the regions. The magnitude of unfavorable management outcomewas highest in the Tigray region, followed by Oromia, SNNP, and Amhara regions. Similar to this finding, a previous systematic review and meta-analysis in Ethiopia discovered that the Tigray region of Ethiopia had the highest prevalence of SSI (40.6%)[53].

In our study, the most commonly reported symptoms of unfavorable management outcome of intestinal obstruction were surgical site infection, pneumonia, septic shock, intraabdominal collection, anastomotic leak, and facial dehiscence, respectively. Similar to the current study, a previous study in Kenya [54], Botswana [55], and Nigeria [49] revealed that surgical site infection was the most common poor surgical outcome, followed by postoperative pneumonia and anastomotic leak. This could be due to improper preoperative and postoperative antibiotic administration [56]. In this regard, different studies have shown that patients with intestinal obstruction should receive preoperative and postoperative antibiotics in the occurrence of perforation [57, 58]. Perioperative antibiotic administration is determined by a variety of factors, including the anatomic region undergoing the specific surgical procedure, the timing of the surgery, the patient’s age, the time of antibiotic administration, the urgency of the procedure, and the availability of the drugs [42, 59]. Most professionals do not follow the guidelines established to prevent infection by taking into account the aforementioned factors. As a result, by following WHO recommendations [60], the burden of SSI and other complications can be reduced.

The present study also demonstrates that there is a significant association between unfavorable management outcome of intestinal obstruction and the duration of postoperative hospital stays, length of illness, comorbidity, dehydration, and intraoperative procedure. In this study, surgically treated patients who stayed in the hospital for more than 8 days after surgery were 9.38 times more likely to experience a poor management outcome than patients who stayed in the hospital for less than 8 days. This outcome was consistent with findings from previous studies in Rwanda [17] and Uganda [61]. This could be because short hospital stays reduce the likelihood of patients acquiring nosocomial infections like hospital-acquired surgical site infection, pneumonia, and deep vein thrombosis [62].

The current study found that patients seeking intestinal obstruction care who arrived later than 24 h were approximately 3.87 times more likely to develop poor management outcomes than patients who arrived early within 24 h. This is in agreement with the studies conducted in Rwanda [17], and Niger [49]. This could be because those who arrived at the hospital early have a lower risk of developing complications such as sepsis and peritonitis, as well as a lower risk of developing gangrenous intestinal obstruction. Furthermore, early and timely intervention for patients increases the likelihood of favorability or early presentation in the case of intestinal obstruction reducing disastrous outcomes, such as a high rate of complications, long hospital stays, and high mortality [45].

In the present study, patients who presented with a comorbid disease were 4.90 times more likely to have unfavorable management outcome of intestinal obstruction compared to those without a co-morbid disease. This finding is consistent with the findings of a study conducted in Turkey [63]. This may be due to the fact that coexisting conditions like diabetes may slow the healing process and raise the risk of postoperative complications like wound dehiscence and infection at the site of the incision [64], which are undesirable surgical management outcome of intestinal obstruction.

Similarly, patients who had dehydration were 6.01 times more likely to have a poor management outcome than those who did not have dehydration. This result is in line with the outcome of a study done in Hong Kong City, China [65].

Finally, in terms of intraoperative procedure bowel, patients who had resection and anastomosis had a 3.85 times greater risk of developing an unfavorable surgical management outcome than patients who did not have resection and anastomosis. This could be due to the fact that resection and anastomosis increase the risk of complications like paralytic ileus, anastomotic leak, and early postoperative adhesion [43].

### Strengths and limitations of the study

This study is the first of its kind in Ethiopia, and it is based on a search for existing and unpublished studies, as well as the use of various perspectives to strengthen the study. However, all of the studies in this systematic review and meta-analysis are cross-sectional. As a result, it is impossible to establish temporal correlations between cause and outcome variables. The majority of the studies included in this evaluation had small sample sizes, which may have an impact on the final estimate. Furthermore, because this meta-analysis included study from only a small portion of Ethiopia, it is possible that the country’s many regions were under-represented. No data are available for Addis Ababa, Harari, Afar, Benshangul Gumze, Dire-Dawa, Gambella, or Somalia, among other regions. As a result, the results might not be representative of the aforementioned regions. Another limitation could be the possibility of missing study due to the inaccessibility of all databases. Having these limitations, we believe this study provides a pivotal data on the magnitude and associated factors of poor management outcome in surgically treated intestinal obstruction patients in Ethiopia important for policy makers.

## Conclusions

In this study, the magnitude of poor management outcome among surgically treated patients was found to be higher in Ethiopia. Tigray had the highest prevalence of unfavorable management outcome, followed by Oromia, SNNP, and Amhara regions. Surgical site infection, pneumonia, septic shock, intraabdominal collection, anastomotic leak, and facial dehiscence were the most commonly reported symptoms of unfavorable management outcome of intestinal obstruction.

The length of postoperative hospital stays, length of illness, comorbidity, dehydration, and intraoperative procedure were significantly associated with unfavorable management outcome of intestinal obstruction. Based on the findings, it is recommended that efforts should be made to reduce unfavorable management outcome of intestinal obstruction. It is also important to note that physicians should diagnose intestinal obstruction early and implement appropriate interventions before the complication occurs. It is also advised to evaluate the comorbidities and give treatment before surgery. Similar to this, it is crucial to administer fluid resuscitation to dehydrated patients to improve surgical management outcomes for patients with intestinal obstruction. Additionally, it is recommended to implement efficient infection prevention measures in hospital settings. Finally, based on this finding, other standard procedures other than resection and anastomosis are recommended.

### Acronyms

SSI (Surgical Site Infection), IO (Intestinal Obstruction), SBO (Small Bowel Obstruction), LBO (large bowel obstruction), SNNP (Sothern nation nationalities and Peoples of Ethiopia), MeSH (Medical Subject Headings), and PRISMA (Preferred Reporting Items for Systematic reviews and Meta-Analyses).

## Electronic supplementary material

Below is the link to the electronic supplementary material.

Additional File 1: PRISMA 2009 Checklist

## Data Availability

The datasets used and/or analyzed during the current study are available from the corresponding author upon reasonable request.
